# Materials for electronically controllable microactuators

**DOI:** 10.1557/s43577-024-00665-1

**Published:** 2024-02-21

**Authors:** Michael F. Reynolds, Marc Z. Miskin

**Affiliations:** Department of Electrical and Systems Engineering, University of Pennsylvania, Philadelphia, USA

**Keywords:** Actuation, Robotics, Microelectromechanical systems (MEMS), Nanoelectromechanical systems (NEMS)

## Abstract

**Abstract:**

Electronically controllable actuators have shrunk to remarkably small dimensions, thanks to recent advances in materials science. Currently, multiple classes of actuators can operate at the micron scale, be patterned using lithographic techniques, and be driven by complementary metal oxide semiconductor (CMOS)-compatible voltages, enabling new technologies, including digitally controlled micro-cilia, cell-sized origami structures, and autonomous microrobots controlled by onboard semiconductor electronics. This field is poised to grow, as many of these actuator technologies are the firsts of their kind and much of the underlying design space remains unexplored. To help map the current state of the art and set goals for the future, here, we overview existing work and examine how key figures of merit for actuation at the microscale, including force output, response time, power consumption, efficiency, and durability are fundamentally intertwined. In doing so, we find performance limits and tradeoffs for different classes of microactuators based on the coupling mechanism between electrical energy, chemical energy, and mechanical work. These limits both point to future goals for actuator development and signal promising applications for these actuators in sophisticated electronically integrated microrobotic systems.

**Graphical Abstract:**

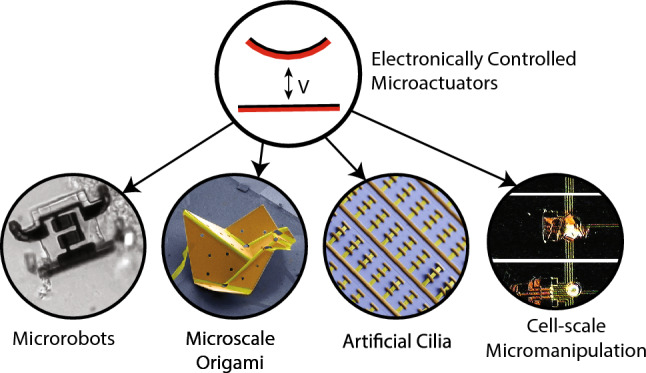

## Introduction

**Figure 1:**
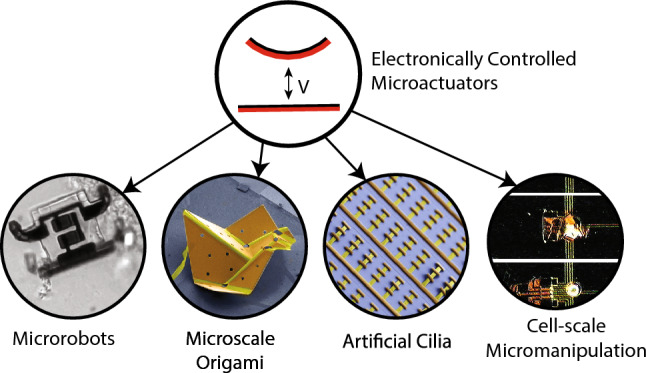
Actuators that respond to electronic control signals yet operate at dimensions under a millimeter have enabled a variety of remarkable applications. Such tiny devices can be used to make microscopic robots,^6^ turn 2D lithographic patterns into controllable 3D origami structures,^7^ pump liquids under user command with electronically controlled cilia,^25^ and manipulate cells and microorganisms with microgrippers.^15^ These applications are evolving rapidly, thanks to electronic control: circuits can be used to generate behaviors that respond to external stimuli^6^ or are reprogrammed on-demand.^26^

In the past 10 years, microactuators have seen significant reductions in accessible length scales (sub 1 µm), control voltages (~1 V), and power consumption (1–10 nW), thanks to a host of new materials. As highlighted in **Figure** **1**, these advances have enabled mechanical systems to easily integrate with the tiny packages of sensors, power, and computation, pointing to a near term future in which autonomous, programmable machines can help shape and control the microworld.

**Figure 2:**
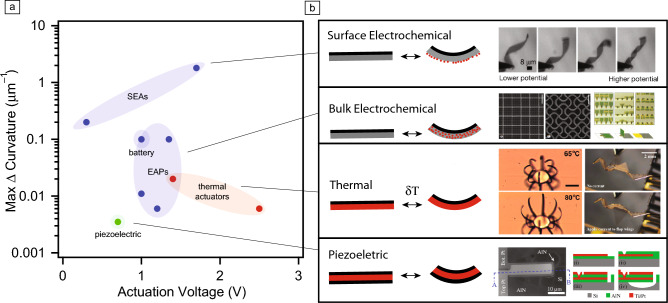
Examples of electronically controlled microactuators. (a) Actuators that operate at low voltage (sub 10 V) and high curvature (>1 mm^−1^) can readily integrate with circuits at the microscale. In recent years, several classes of actuators have emerged that meet these demands, spanning operating voltages from ~100 mV to 3 V and curvatures up to 1 µm^−1^. Broadly, actuators can be classified as electrochemical (bulk^4,5,13,14^ and surface^7,8^), thermal,^2,3^ and piezoelectric.^9^ (b) Examples of each class of actuators have been demonstrated at the microscale, including surface electrochemical actuators^7^ (top panel), bulk electrochemical actuators using the lithiation of silicon to buckle microscale beams^14^ or the charging of polymer layers to control microgrippers^4,5,13^ (second from top), thermal actuators for microscale grippers^1^ and origami^2^ (second from bottom), and nanometer-thick aluminum nitride piezoelectric actuators^8^ (bottom panel).

Arguably, the dominant actuation approach at the microscale is to use bending/folding mechanisms for moving parts.^1^ Bending avoids issues with stiction, is well suited to the two-dimensional (2D) patterning used in lithographic fabrication, and, because pure elastic bending is essentially scale invariant, allows designs to be scaled up or down in size by proportionally altering all the dimensions. With an eye toward microsystems, we focus here on bending actuators where the operating voltage is under 10 V and the curvature is larger than 1 mm^−1^ (**Figure** **2**) as actuators outside of these bounds require nontrivial voltage conversion or operate at too large of a curvature to be useful in a submillimeter machine. Demonstrated electronically controlled bending microactuators that fall within these constraints operate via three mechanisms: thermal,^2,3^ electrochemical,^4–8^ and piezoelectric.^9^

An emerging question for this field is how to quantify actuator performance at the microscale. Given the distinct physics of tiny machines, it stands to reason that microactuators should be gauged by figures of merit that are different from those used to describe their macroscale cousins. For instance, power-to-weight ratio is not useful in a world where gravitation forces are negligible compared to drag and surface forces. Further, for many microactuators, energy consumption scales with area, making areal figures of merit more informative than volumetric ones.

This article seeks to establish a new framework for comparing microscale actuators, giving rules that are useful in selecting a given actuator for a given task and for quantifying progress as new materials for microactuators come into focus. We argue that key figures of merit at the microscale include durability, the force normalized by width-to-length ratio (force per square), efficiency of work, and response time as these considerations are well suited both to the physics of the microworld and the design considerations of microfabrication. We also note that these metrics are not fully orthogonal: for instance, in many actuators, we find efficiency and strain are fundamentally coupled as are the force output and response time. These results show where improvement could be gained, and where current actuators are near fundamental bounds.

## Bending microactuators

### Thermal actuators

Thermal microactuators achieve bending by heating layered stacks of two or more materials with different thermal-expansion properties. They can be controlled electrically via Joule heating or externally by laser illumination. Thermal microactuators have many attractive features, including fast actuation, large force outputs, relatively low driving voltages (1–10 V), and repeatable bending over many cycles. At the submillimeter scale, prior works have demonstrated electrically controlled thermal actuation with strains up to ~2% by leveraging thermal phase transitions^2^ and shape-memory behavior by reflowing polymers during actuation.^3^

One major challenge for using thermal actuators, especially when used in autonomous microsystems such as robots, is their large power consumption. Previous examples of microactuators at the 100 µm to 1 mm scale driven by Joule heating require ~1 mA currents and powers around 1 mW. These large electrical requirements stem from heat lost to the environment: dimensional analysis indicates that the power lost to the surrounding environment is $\sim\upkappa L\Delta T$, where $\upkappa$ is the thermal conductivity of the surrounding medium, Δ*T* is the difference in temperature between the actuator and its environment, and *L* is the length of the actuator. For submillimeter actuators in air ($\upkappa$* ≈* 10^–2^ W/mK), the minimum power required to heat the actuator by 10 K is about 100 µW, a challenging constraint for an untethered microrobot. While there is significant ongoing progress on batteries for small-scale robots (as highlighted by the article by Schmidt and Zhu in this issue^10^), the best existing batteries at any scale have a volumetric energy density of about 1000 Wh/L,^11^ giving a 100 µm cubic battery enough energy to power fewer than 100 actuation cycles. Even photovoltaic power, which scales as length squared, would be insufficient to power a thermal actuator: a 100-µm-square silicon photovoltaic with a 10% power-conversion efficiency produces about 1 µW of power in full sunlight, two orders of magnitude too low to drive actuation.

**Figure 3:**
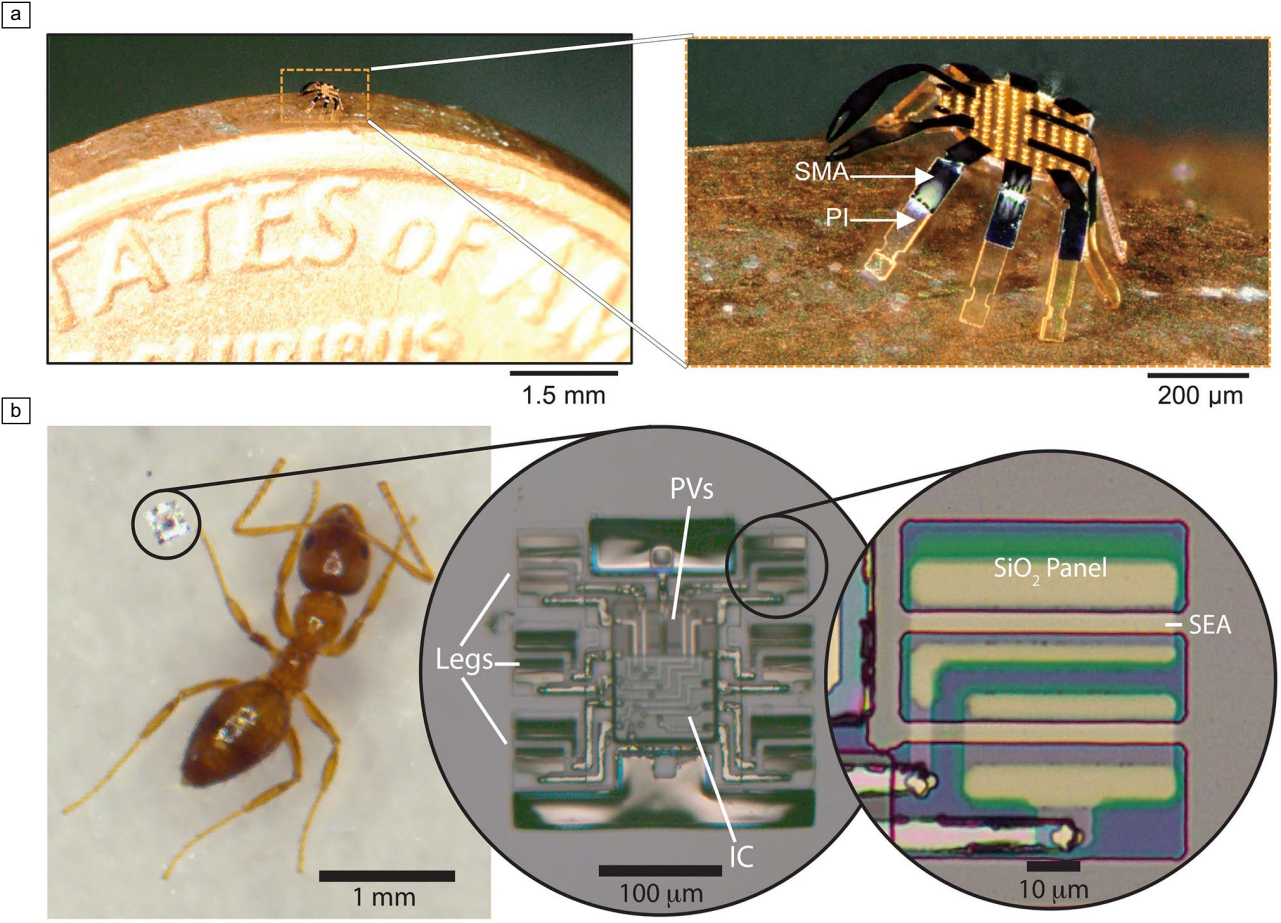
Examples of microrobots with bending microactuators. (a) Microrobots with thermal microactuators made with nitinol shape-memory alloys (SMAs).^12^ Direct laser actuation heats the hinges, causing them to bend and the robots to walk. These microrobots walk on land at speeds close to a body length per second and can be tracked with onboard retroreflectors. (b) Microrobots with surface electrochemical actuators and onboard digital control electronics.^27^ Both the legs and the circuit on these robots are powered by light. The onboard microelectronic circuit generates clock signals to drive the legs and set the gait of the robots. These microrobots operate in aqueous environments, move at close to 0.1 body lengths per second, and can change behavior in response to optically delivered commands. PVs, photovoltaics; IC, integrated circuit; SEA, surface electrochemical actuator.

A clever workaround to thermal actuation’s high power demand was demonstrated in Han et al. and is shown in **Figure** **3**a: rather than use onboard power, a targeted laser directly heats the actuator, enabling untethered submillimeter robots that walk on land.^12^ The authors maintained the optical power link over long ranges by integrating retroreflective materials on the robot’s body. Indeed, thermal actuation is well suited to terrestrial microrobots because walking on a surface demands forces large enough to overcome adhesion, while legged locomotion requires high durability, two key features of this actuator class.

### Electrochemical actuators

Several groups have demonstrated microactuators that bend in response to electrochemical charging. These actuators operate in conductive solutions, and bending is controlled by transferring charge between the solution and the actuator. Demonstrated examples include conductive (conjugated) polymers,^5,6,13^ metals,^7,8^ and battery materials.^14^

Conjugated polymers electrochemical actuators leverage redox reactions of the polymer with ions in the solution. These reactions generate a strain in the conductive polymer layer which, when stacked with a passive layer, generates bending.^15^ The first work on these polymer actuators used electrodeposited polypyrrole (Ppy) on gold to demonstrate micron-thick bilayer actuators with ~0.1 µm^−1^ curvature changes and forces of about a micronewton per square.^3^ Since then, Ppy has been used to make robotic grippers with multiple joints,^16^ microactuators with onboard strain sensing,^17^ and ciliated surfaces for pumping fluid.^18^ More recently, PEDOT:PSS microactuators have been demonstrated.^5^ Although PEDOT:PSS exhibits smaller strains than Ppy actuators, it can be spin-coated onto a variety of surfaces, simplifying fabrication.

Conjugated polymer microactuators are frequently used in microrobotic systems for their combination of high forces and curvatures. Their primary drawbacks are related to speed and durability. The maximum frequency of actuation is about 1 Hz,^4,17^ limited by mass transport.^4^ Ppy actuators also fail after 1000–10,000 cycles due to delamination between the active layer and the metal film onto which it is deposited.^4,15^ Recent works have demonstrated actuation through at least 1000 cycles without delamination by adding layers to move the neutral axis of bending closer to the metal/Ppy interface, decreasing the strain this interface experiences.^17^

While previously demonstrated CP microactuators require an electrolyte, they operate in biologically relevant saline solutions, making them promising actuators for biomedical applications.^18^ Future microactuators could leverage macro- and milliscale trilayer actuator designs that include polymer electrolyte membranes to operate outside fluid environments.^19–23^

Battery materials are known to expand dramatically when charging, giving an interesting route to microactuators with high strains. In particular, Xia et al. demonstrated microscale beam bending via lithiation of a microstructured silicon anode.^14^ By leveraging the >300% expansion of silicon during lithiation, they demonstrate a variety of electrically controlled bending and buckling structures and programmable metamaterials. These structures produce large bending forces and, despite being 100’s of nm thick, bend to 10–100 µm radii of curvature (${R}_{c}$).

Although large bending forces and curvatures recommend battery materials as microactuators, lithiated silicon also has several downsides. First, the actuation response time is on the order of minutes, limited by the diffusion of lithium into the silicon. In principle, this could be improved by using thinner active layers, trading force output for response time. Second, the expansion is so dramatic that repeated charging and discharging fracture the silicon and degrade the actuation response over just a few cycles. Indeed, expansion and resultant mechanical fracture are well-known issues with silicon battery anodes.^24^ Actuators where silicon is the only active material also require a solution of lithium salt to operate in, though future battery-material-based actuators could include anode/solid electrolyte/cathode trilayer stacks, creating fully self-contained microactuators.

Surface electrochemical actuators (SEAs) leverage surface chemistry of metals to drive bending at the micron scale.^7,8^ SEAs consist of ultrathin platinum capped on one side by a passive layer with a total stack thickness of about 10 nm. In aqueous environments, surface electrochemistry at the platinum surface—either adsorption of ions on the platinum^7^ or oxidation of the platinum surface^8^—generate surface stresses that cause bending. SEAs exhibit several unique features: they bend to curvatures of about ~1 µm^−1^, operate with approximately nW input power, exert forces of about 1–10 nN, exhibit frequencies between 10 and 100 Hz (limited by fluid drag), and actuate repeatedly over thousands of cycles. The same structures also function as chemically responsive microactuators in air.^25^

These actuators demonstrate higher curvatures and operation frequencies than other electrochemical microactuators at appreciably lower voltages and powers; the tradeoff for these benefits is a comparatively low force output. Because SEAs rely on surface stresses for actuation, gains in force from making the actuator thicker are limited: force increases linearly with thickness, *t*, and radius of curvature decreases as *t*^−2^. Similar to other electrochemical microactuators, SEAs’ operation is currently limited to aqueous electrolyte environments. Ongoing work related to SEAs is exploring microactuators that use other metals with electrochemical activity or integrating electrolytes into a single packaged actuator.

Nonetheless, the low power requirements, relative ease of fabrication and integration with microelectronics, and durable actuation over many cycles make them a model actuator for microrobotic systems. The fact that SEAs actuate at hundreds of millivolts allows them to trivially integrate with semiconductor electronics, because the same voltage scales are required to drive a transistor into saturation. In turn, SEAs have been used to build electronically integrated versions of microscale origami structures,^8^ micro-cilia arrays,^26^ and microscopic robots powered and controlled with onboard circuits.^7,27^

One of the most sophisticated examples of electronics integration was demonstrated by Reynolds et al. who constructed fully autonomous robots, shown in Figure 3b.^27^ Each machine, powered by onboard solar cells, can walk across a substrate using onboard semiconductor electronics to control gait. Beyond walking, robots within this work could alter behavior on command: a user can send instructions as time varying optical signals, which the robot decodes and implements by speeding up its gait cycle. Ongoing work seeks to extend these capabilities further, incorporating more sophisticated control electronics such as microprocessors, sensors, and memory.^28^

### Piezoelectric actuators

At the millimeter scale and larger, piezoelectric actuators are ubiquitous because of their repeatable actuation, high-frequency response, and high efficiency. Applications include insect-scale walking and flying robots.^29–33^ These systems typically use lead zirconate titanate (PZT) and achieve bending radii approaching 1 mm with micron-thick layers operating 10 s of volts.^33,34^ However, making bending piezoelectric actuators at the micron scale is a challenge. Piezoelectric materials generate relatively low strains, from 0.01 to 1 percent. To achieve submillimeter radii of curvature with a multilayer stack that includes a piezoelectric, top, and bottom electrode layer, the active material must have a thickness of 10–100 nm. This imposes stringent materials constraints for growth because the film must be crystalline and deposit on ultrathin metal electrodes to achieve actuation. One example of piezoelectric actuators with submillimeter bending consists of an aluminum nitride piezoelectric layer and platinum electrodes, all less than 30 nm in thickness.^9^ Because they are so thin, these microactuators operate at <1 V, bend to about 300 µm radii of curvature and exhibit fast (up to megahertz) actuation.^35^

Despite relatively smaller displacements compared to electrochemical and thermal actuators, piezoelectric actuators’ high operating frequencies, high efficiencies, and low power consumptions make them promising candidates for a variety of microrobotic applications. For instance, because they operate in air by design, they are a natural fit for terrestrial robots. Likewise, larger (>100 μm) microscopic robots could use high-frequency operation to compensate for low displacement, allowing the robot to take fast, small steps to achieve reasonable speeds.

## Tradeoffs for microactuator performance

### Strain, efficiency, and durability

**Figure 4:**
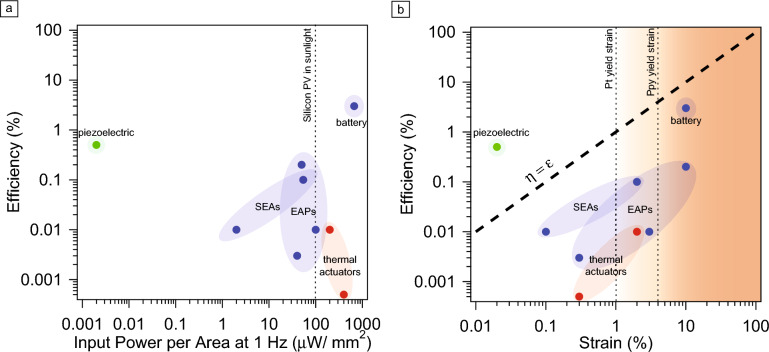
(a) Efficiency versus power consumption per square millimeter for actuators operating at 1 Hz. The dotted line shows the approximate power per millimeter square for a silicon photovoltaic (PV) in bright sunlight (given by 1 mW/mm^2^ incident light intensity and assuming PV efficiency is about 10%). Even for microactuators with comparable efficiencies, power consumption can vary over almost six orders of magnitude. (b) Efficiency versus strain for microactuators shows a general relationship between the two: more efficient actuators operate at higher values of strain. In the case of electrochemical actuators, this result can be rationalized by looking at the dominant scaling behavior for electrical and mechanical energy. Moreover, if efficiency and strain scale together, then there are fundamental limits on actuator performance set by the elastic limits of the constituent materials. Indeed, vertical lines show where constituents for electrochemical actuators would begin to fail, indicating that within this class, further improvements in efficiency could be impossible without material innovation. Data are drawn from the following references: electrochemical bulk,^4,5,13^ electrochemical surface,^7,8^ battery,^14^ thermal,^2,3^ and piezoelectric.^9^ SEAs, surface electrochemical actuators; EAPs, electroactive polymers.

Energy storage is difficult in microsystems due to the small available volume, and instead actuators are often connected to continuous power sources to achieve long-term operation. Consequently, the efficiency or proportion of power utilized for work can often be less important than the nominal power draw. Indeed, **Figure** **4**a shows a plot of power required for actuation against efficiency, *η*, which we define as the ratio of mechanical work necessary to deform the actuator to a given deflection to the electrical energy expended during deformation. We note that although many actuators have comparable efficiencies, the actual power input can differ by several orders of magnitude. For instance, compare SEAs and thermal actuators. Both are nearly equal in efficiency but differ dramatically in nominal power: SEAs consume nW, whereas thermal actuators consume >100 µW. As a result, it is straightforward to integrate SEAs with an onboard power source in a microrobot, while, as noted earlier, it is difficult to do the same with a thermal actuator.

Although efficiency may not be sufficient to determine which actuator is well suited to an application, it can still be used to describe fundamental limits of actuator performance. For instance, Figure 4b shows the efficiency for each microactuator plotted against the strain due to bending, $\upepsilon \approx {{R}_{c}}^{-1}t$. Strains vary from 10^–4^ to 10%, and the data show a roughly linear relationship between strain and efficiency for electrochemical and thermal actuators, with piezoelectric actuators as a distinctly different class.

Because the mechanical work for any bending actuator, regardless of mechanism, is given by ${U}_{m}\sim {E}_{y }twL{\upepsilon }^{2},$ where ${E}_{y}$ is the Young’s modulus and $w$ is the width, different relationships between efficiency and strain must arise because of different scaling laws linking electrical work ($QV$) and deformation.

One possibility is that the strain is proportional to electrical work ($QV\propto \upepsilon$), leading to an efficiency that is also proportional to strain. This is the most common case for microactuators, applying to thermal, dielectric electroactive polymers, and SEAs. For instance, dielectric electroactive polymers actuators operating in the elastic limit experience a strain $\upepsilon =QV/{E}_{y}twL$, where *t* is the thickness of the polymer layer. SEAs also follow this trend: *QV* is proportional to the surface stress, which in turn is proportional to strain. Although thermal actuators do not rely on charge storage, a similar argument leads to the same scaling. For a thermal actuator, the minimum energy input to drive bending is linear in Δ*T*. Because strain and temperature are proportional in thermal actuation, the input energy scales with strain and, by extension, so does the efficiency.

A second possibility, displayed by piezoelectric actuators, is that $QV\propto {\upepsilon }^{2}$. A piezoelectric actuator behaves similar to a capacitor in the electrical domain, while the built-in electrical polarization of the material causes strain to scale linearly with applied voltage. In this case, the efficiency is a strain-independent material parameter (i.e., the electromechanical coupling). This leads to actuators, including the green point on Figure 4b, with high efficiencies despite having relatively low strains.

The previously discussed analysis points to a simple principle for improving the efficiency of many actuators: increase strain. However, the yield strains of the actuator’s constituent materials set a limit on how much can be gained by this approach as past this point actuators fail over repeated cycling. Figure 4 indicates yield strain for two common microactuator materials, platinum and polypyrrole, showing that many actuators are already operating at, and in some cases past, their yield strain limit. Indeed, actuators start to fail in these high-strain cases: SEAs achieve higher curvatures and efficiencies for initial cycles when driven via oxidation instead of surface adsorption, but decrease in actuation amplitude by a factor of two over several thousand cycles; polypyrrole actuators with metal layers delaminate over about 1000 cycles; and, most drastically, lithiated silicon, while achieving more than 10% strain in a hard material, fails over about 10–100 cycles. In general, an actuator’s best balance between efficiency, actuation amplitude, and repeatability over many cycles requires operating just below the yield strain of their lowest yield strain material. Achieving a higher efficiency without sacrificing durability would require new materials with larger elastic windows, stronger coupling between electrical and mechanical energy, and/or lower operating voltages.

### Force and response time

**Figure 5:**
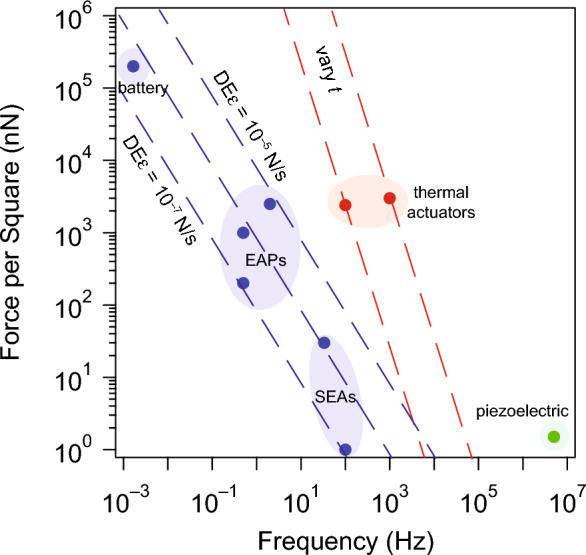
Force per square against actuator response time shows that microactuators can operate over a wide range, but evidently face a performance tradeoff. An engineer can currently choose between a fast, weak actuator or a slow strong one, but no actuator achieves both high force and fast response. For thermal and electrochemical actuators, these limits arise from transport constraints: an actuator needs to reach thermal (chemical) equilibrium to impart force. Future work could engineer these transport properties, thereby improving response time. Data are drawn from the following references: electrochemical bulk,^4,5,13^ electrochemical surface,^7,8^ battery,^14^ thermal,^2,3^ and piezoelectric.^9,35^ SEAs, surface electrochemical actuators; EAPs, electroactive polymers.

Response time and force are critical variables for any actuator and **Figure** **5** shows they vary broadly for microactuators. The actuators surveyed here differ by almost ten orders of magnitude in response time and six orders of magnitude in force, enabling a wide range of possible applications. Yet there is also an evident tradeoff between the two: actuators that supply higher forces also tend to work at slower speeds.

For both thermal and electrochemical actuators, this dependence arises because relaxation time and force both scale with the thickness of the actuator: thicker actuators can supply more force, but they also take longer to equilibrate. Specifically, for a bending actuator, the force per square scales quadratically with thickness, $\sim{E}_{y}{t}^{2}\upepsilon$ while thermal (electrochemical) actuators have response times that scale linearly (quadratically) with thickness. Specifically, the thermal relaxation is set by the rate of passive cooling to the environment as $\uptau \sim \frac{CLt}{\upkappa },$ where *C* is the volumetric specific heat capacity, *L* is the lateral dimension of the actuator, and $\upkappa$ is the thermal conductivity of the environment, while chemical equilibration depends on diffusion of chemical species into the actuator material giving $\uptau \sim {t}^{2}/D,$ where *D* is the diffusion coefficient.

The fact that few high-speed, high-force actuators exist poses interesting design challenges for microrobotics. For instance, walking robots that operate in air require larger force scales to overcome adhesive forces. Yet given the available actuator choices, a robot of the same size could potentially walk faster through water, despite viscous drag forces, thanks to faster actuators. Future work may be needed to mitigate this tradeoff, potentially improving interlayer transport of electrochemical actuators or altering the heat capacity or conductivity of thermal actuators to increase the speed.

## Outlook for microactuators

The features shared by these material platforms point to a bright future for microactuators. First, the actuators reviewed here are all made from materials that can be processed massively in parallel with lithographic techniques and deposited directly on top of prefabricated electronics. Combined with the fact that they use actuation voltages low enough to integrate directly with microelectronics, these actuators point to a possible future where mechanical elements can be seamlessly integrated with semiconductor electronics, enabling us to build tiny robots as easily as we build circuits.

A core challenge in realizing this vision, at least in the near term, will be establishing best practices for fabrication and integration. Integrating actuators with electronics can still be difficult because of compatibility issues that arise in microfabrication. The works listed here demonstrate ways forward for each actuator class, but further exploration in this space could lift more fabrication constraints, enabling actuators to be added to broader classes of microsystems.

Achieving these technical goals will help sustain the recent advances in small-scale robotics. Actuators and electronics processed lithographically already enable robot swarms of nearly 10,000 agents to be made massively in parallel and deployed all together.^7^ By integrating onboard semiconductor circuits to control actuation, microroboticists are now able to shift away from purely mechanical solutions to locomotion toward programmable, electronic ones.^27^ Finally, the overall cost of production for each machine remains at fractions of a penny even when complex circuits are included, inviting new applications for robots too small to see by eye in microfluidics, drug delivery, manufacturing, and materials science.

## Data Availability

Not applicable.
